# Empathic Traits Modulate Oscillatory Dynamics Revealed by Time–Frequency Analysis During Body Language Reading

**DOI:** 10.3390/brainsci15070673

**Published:** 2025-06-23

**Authors:** Alice Mado Proverbio, Pasquale Scognamiglio

**Affiliations:** 1 Neuro-Mi, Milan Center for Neuroscience, University of Milano-Bicocca, 20126 Milan, Italy; 2 Master’s Program in “Human-Centered Artificial Intelligence” HCAI, Department of Philosophy, University of Milan, 20122 Milan, Italy; pasqualescognamiglio2002@gmail.com; 3 Cognitive Electrophysiology Laboratory, Department of Psychology, University of Milano-Bicocca, Piazza dell’Ateneo Nuovo 1, 20162 Milan, Italy

**Keywords:** EEG, time frequency, alpha, mu suppression, attention, individual differences, mirror neurons, empathy

## Abstract

Empathy has been linked to enhanced processing of social information, yet the neurophysiological correlates of such individual differences remain underexplored. **Objectives**: The aim of this study was to investigate how individual differences in trait empathy are reflected in oscillatory brain activity during the perception of non-verbal social cues. **Methods**: In this EEG study involving 30 participants, we examined spectral and time–frequency dynamics associated with trait empathy during a visual task requiring the interpretation of others’ body gestures. **Results**: FFT Power spectral analyses (applied to alpha/mu, beta, high beta, and gamma bands) revealed that individuals with high empathy quotients (High-EQ) exhibited a tendency for increased beta-band activity over frontal regions and markedly decreased alpha-band activity over occipito-parietal areas compared to their low-empathy counterparts (Low-EQ), suggesting heightened attentional engagement and reduced cortical inhibition during social information processing. Similarly, time–frequency analysis using Morlet wavelets showed higher alpha power in Low-EQ than High-EQ people over occipital sites, with no group differences in mu suppression or desynchronization (ERD) over central sites, challenging prior claims linking mu ERD to mirror neuron activity in empathic processing. These findings align with recent literature associating frontal beta oscillations with top-down attentional control and emotional regulation, and posterior alpha with vigilance and sensory disengagement. **Conclusions**: Our results indicate that empathic traits are differentially reflected in anterior and posterior oscillatory dynamics, supporting the notion that individuals high in empathy deploy greater cognitive and attentional resources when decoding non-verbal social cues. These neural patterns may underlie their superior ability to interpret body language and mental states from visual input.

## 1. Introduction

Understanding others’ minds and emotions is foundational to human interaction. Empathy and Theory of Mind (ToM) are central to this process, allowing individuals to resonate with others’ affective states and infer their mental contents. These abilities rely on partially overlapping, yet functionally distinct, neural systems: while affective empathy engages regions such as the anterior insula and anterior cingulate cortex [1,2], ToM involves more distributed cognitive mechanisms. A growing body of evidence has highlighted the role of trait empathy, a stable disposition to resonate with others’ emotions and understand their perspectives [3,4], in shaping how these social cognitive systems are recruited. Notably, trait empathy has been shown to enhance neural sensitivity in tasks involving belief attribution [5] and to increase interindividual phase synchrony during joint attention [6], supporting the idea that dispositional empathy modulates both affective and cognitive components of the social brain.

Despite this, it remains unclear whether trait empathy can be indexed by specific electrophysiological markers, especially during nonverbal social inference tasks such as body language comprehension. Here, we address this gap by examining whether EEG oscillatory activity—specifically, patterns in the alpha (8–13 Hz), beta (15–30 Hz), and mu (sensorimotor 8–13 Hz) bands—differs between neurotypical individuals with high versus low trait empathy during a gesture-based mental state inference task.

### 1.1. Study Aim and Hypotheses

The present study aimed to test whether oscillatory dynamics in EEG recordings reflect individual differences in trait empathy during the visual interpretation of gestures and body postures. Specifically, we hypothesized that high-empathy individuals would exhibit (a) reduced occipito-parietal alpha power, reflecting greater attentional engagement [7,8], (b) increased frontal beta activity, associated with sustained attention and mental state attribution [9], and, possibly, (c) stronger suppression of the mu rhythm, indicative of heightened sensorimotor resonance [10]).

### 1.2. Background and Theoretical Framework

Previous studies suggest that mu rhythm suppression (also known as event-related desynchronization, ERD) during action observation is enhanced in individuals with higher empathy. For example, stronger mu suppression has been associated with higher perspective-taking abilities [11], greater responsiveness to emotionally charged stimuli [12], and enhanced neural sensitivity to social touch [13]. These findings point to a potential link between sensorimotor resonance and empathic processing. However, this association remains contentious. Several studies have failed to replicate these effects. For instance, DiGirolamo et al. [14], in a large-scale study, found no relationship between trait empathy and mu suppression, suggesting that mu rhythms may reflect transient attentional states rather than stable empathic traits. Similarly, Goodarzi et al. [15] reported null results in a clinical sample, raising questions about the generalizability of previous findings. The difficulty in distinguishing mu from occipital alpha activity—given their spectral overlap and spatial proximity—further complicates interpretation [16].

This ongoing debate underscores the need for more nuanced approaches that take into account both trait-level variability and state-dependent modulations of neural activity, particularly during tasks that engage implicit social cognition through nonverbal cues.

### 1.3. Methodological Approach and Rationale

To address these issues, we employed a complementary analytic strategy that combined traditional Fast Fourier Transform (FFT) with high-resolution time–frequency decomposition using Morlet wavelets. FFT enabled the identification of stable, frequency-specific power differences, particularly in the alpha/mu and beta bands [17,18,19,20], while wavelet analysis allowed us to capture transient, non-phase-locked oscillatory dynamics with precise temporal resolution—particularly useful for studying neural responses during brief, socially salient events. The task involved visual interpretation of bodily gestures and postures to infer mental states, a paradigm previously shown to engage empathy-related processes. In a related ERP study on body language comprehension [21], participants with higher trait empathy exhibited faster behavioral responses and stronger frontal N250 and N400 components. These ERP effects suggest early and sustained socio-emotional sensitivity, which we hypothesized would also manifest in the frequency domain. By integrating both frequency-based and time-resolved EEG analyses, our study aimed to provide a comprehensive assessment of whether and how oscillatory brain activity reflects empathic trait dispositions, moving beyond simplistic models and addressing the complex interplay between attention, affect, and social cognition.

## 2. Materials and Methods

### 2.1. Participants

Thirty healthy male and female university students ranging in age from 20 to 31 years (mean age = 23 years, SD = 2.9 years) were included in the study. EEG data from 1 participant (male) were disregarded due to an abundance of EEG artifacts. The final sample consisted of 14 males and 15 female participants, with a mean age of 23.4 years. Fifteen participants constituted the High-EQ group (mean age = 23.07, SD = 2.28), and fourteen participants formed the Low-EQ group [mean age = 23.78, SD = 3.5] (Table 1). The two experimental groups were classified according to their empathy quotient, assessed using the Italian-language version of the *Empathy Quotient* [22], which was administered to participants subsequent to EEG recordings. This questionnaire is derived from the original *Empathy Quotient* by Baron-Cohen et al. [23], as it was adapted and validated for the Italian population while maintaining the theoretical framework of the original scale.

Participants were matched for socio-cultural status and were all native Italian speakers. Their lateral preference was assessed through the administration of the Edinburgh Inventory. All participants had normal or glass-corrected vision and normal hearing. No participant suffered or ever suffered from neurological or psychiatric disorders (including dyslexia, alexia, autism, ADHD, depression, or personality disorders). All participants provided written informed consent and were unaware of the research purpose. The study lasted approximately 2 h, encompassing breaks, training, and administering the questionnaire. Questionnaire administration followed EEG recording, so as not to induce psychological bias. Participants voluntarily offered their participation or received academic credits. The Ethics Committee of the University of Milano-Bicocca (protocol number 0000273/14/08012014) approved the project.

### 2.2. Stimuli

**Figure 1:**
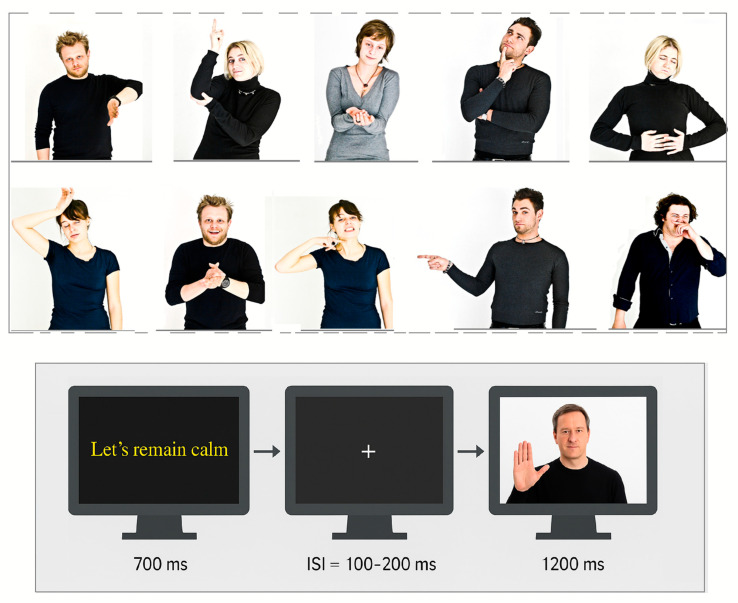
(**Top**) Examples of body language displays (congruent with their captions). From left to right (first row): Let us put this aside; May I have a word? A little something, please; Let me think about it; I am full, I have eaten too much. *Second row:* What an unbearable heat; Oh, how wonderful! I’m going to kill you! It was him! Such an intolerable smell! The captions were originally presented in Italian. The complete set of stimuli is available via the Bicocca Open Archive Research Data, DOI: 10.17632/vwwmmd99r3.1. Informed consent and release forms were obtained from all individuals photographed. (**Bottom**) Temporal layout of the experimental procedure.

The stimuli comprised 200 images selected from a validated set of pictures designed to assess nonverbal behavior [24]. Each color photograph depicted half of the body of a male or female actor, attired in a black sweater and captured against a white background (Figure 1). The set included six distinct actors (three males and three females) performing 187 unique gestures, categorized into one of three types: iconic (visual representations of referential meaning), deictic (gestures indicating real, implied, or imagined persons, objects, or directions), or emblematic (symbolic and conventional gestures conveying standardized meanings, such as a thumbs-up gesture to denote ‘good’). The stimuli were 371 × 278 pixels in size and had an average luminance of 13.66 cd/m^2^. Each gesture was accompanied by a brief verbal label, presented in yellow Times New Roman font for 700 ms at the center of the screen, followed by an inter-stimulus interval (ISI) ranging from 100 to 200 ms before the corresponding image appeared. The outer background of the display was light gray.

### 2.3. Procedure

Participants were comfortably seated in a dark, acoustically and electrically shielded cubicle, positioned 100 cm from a high-resolution screen. They were instructed to maintain their gaze on a central fixation cross on the screen and to minimize eye or body movements throughout the session. The task consisted of deciding whether the gesture and its corresponding caption were congruent and also involved perceiving pictures in which faces were obscured. The data processed here only concern the recognition of body language in congruent displays provided with appropriate facial expressions.

### 2.4. EEG Recordings and Analysis

Scalp EEG was recorded from 128 Ag/AgCl electrodes positioned following the 10–5 International placement system, using *ANT Neuro Waveguard* high-density caps designed for optimal spatial coverage and signal fidelity. Horizontal and vertical electro-oculograms (EOG) were simultaneously recorded. Signals were referenced to the averaged mastoids, with electrode impedance maintained below 5 kΩ. The EEG and EOG signals were digitized at a sampling rate of 512 Hz using the Cognitrace system (ANT Software, 4.10.1 version), and amplified with an online bandpass filter set between 0.016 and 70 Hz. EEG epochs time-locked to stimulus onset were extracted from −100 ms to 1200 ms and processed offline using the EEProbe system (ANT Software). Trials containing EEG activity exceeding ±50 μV were automatically rejected prior to averaging. The overall percentage of rejected trials ranged from 5% to 8%. Automated artifact correction procedures (e.g., ICA or ASR) were employed only in rare instances, specifically when aiming to retain data from participants with excessive noise that would otherwise warrant exclusion. Notably, no such cases occurred in the present sample.

FFT analysis was applied to ERP signals reflecting perception of congruently labelled body language displays during a task involving the understanding of body language meaning [21]. In general, to characterize frequency-specific neural dynamics associated with stimulus processing, a FFT power spectrum analysis was applied to ERP epochs extending from 150 ms to 800 ms post-stimulus onset. This time window was selected to exclude early sensory components and focus on sustained cognitive and sensorimotor activity. Spectral decomposition was conducted to quantify oscillatory power within four canonical EEG frequency bands: alpha and mu rhythms typically implicated in attentional and sensorimotor processes; low beta β1 and high beta β2, both of which are often associated with motor preparation and cognitive control; and gamma, a high-frequency range linked to integrative cortical processing and perceptual binding. For each trial, mean spectral power was computed within these bands and subsequently averaged across trials to yield robust estimates of task-related neural oscillatory activity. To examine the temporal evolution of frequency-specific neural activity with high time–frequency resolution, time–frequency decomposition was performed using wavelet analysis on EEG epochs ranging from −100 ms to 800 ms relative to stimulus onset. This approach allows for precise tracking of oscillatory dynamics over time [25,26,27], particularly in the lower alpha band. Spectral power was computed across five narrow frequency bins within the alpha range, enabling a fine-grained characterization of alpha sub-band modulations potentially linked to distinct cognitive processes such as expectancy, attention, and early post-stimulus processing. Delta and theta bands were excluded from analysis, as the temporal windows employed were optimized for capturing transient, event-related responses to visual stimuli. Furthermore, the analysis focused on alpha, mu, and beta rhythms, which are robustly linked to sensorimotor mirroring and empathic processing.

FFT mean spectral power was extracted within four canonical EEG frequency bands: 8–13 Hz (alpha/mu), 15–24 Hz (low beta, β1), 25–30 Hz (high beta, β2), and 38–42 Hz (gamma). An effective NFFT of 512 was applied per epoch, yielding a frequency resolution suitable for short-duration EEG segments. Zero-padding was applied to extend the 333-sample epochs to 512 points prior to FFT computation. Multitaper methods (e.g., Slepian tapers or DPSS) were not used (as featured by the ASA 4.10.1 system).

Time–frequency decomposition was performed using Morlet wavelets with 6 cycles, a frequency resolution of 0.25 Hz, across five narrow alpha sub-bands (8–13 Hz in 1-Hz bins); power estimates were baseline-corrected relative to the –100 to 0 ms pre-stimulus interval. While both FFT and wavelet analyses were conducted and showed converging results, we ultimately deferred to the wavelet-based findings given their greater sensitivity to the non-stationary, time-resolved dynamics of mu and alpha activity. Relevant electrodes for time–frequency analysis were: Fp1, Fp2, AFp3h, AFp4h, F3, F4, C3, C4, CCP1h, CCP2h, CP1, CP2, P3, P4, PO3, PO4, O1, and O2.

Multifactorial repeated measures of analyses of variance (ANOVAs) were applied to EEG power values, using a between-subject factor of empathy group (Low-EQ and High-EQ) and the within-subject factors electrode (depending on the EEG rhythm) and hemisphere (Left and Right). Multiple post hoc comparisons of means were performed by means of Fisher’s tests. To address potential violations of the sphericity assumption inherent to repeated-measures ANOVA, we applied Greenhouse–Geisser correction to all relevant analyses. When the assumption of sphericity was violated, as indicated by Mauchly’s test, Greenhouse–Geisser correction was applied to adjust the degrees of freedom and control for inflated Type I error rates. All reported *p*-values reflect this correction. Partial eta squared values were also computed to provide an estimate of the effect size for each factor in the analysis.

Isocolor topographical maps were performed by plotting surface potentials recorded during the latency range relative to the main ERP components of interest. Spectral power data (µV^2^/Hz), obtained through Morlet wavelet transformation, were analyzed using a repeated-measures ANOVA with Empathic Trait (Low-EQ and High-EQ) as a between-subject factor, and EEG band (8–9 Hz, 9–10 Hz, 10–11 Hz, 11–12 Hz, 12–13 Hz) and Scalp Area (central: C3/4, CCP1h/2h; occipital: PO3/4, O1/2) as within-subject factors. Additional post hoc analyses were conducted where appropriate, including the use of linear mixed-effects models, non-parametric permutation testing, and False Discovery Rate (FDR) correction for multiple comparisons. These procedures were applied to relevant effects observed in the FFT beta power analysis and in the wavelet-based examination of mu and alpha band activity

## 3. Results

### 3.1. Behavioral Data

No significant effect of group was found for accuracy data (Low-EQ = 86.7%, SD = 0.01; High-EQ = 87.6%, SD = 0.01). A Welch’s unequal variances two-sample *t*-test was conducted to evaluate whether mean response times (RTs) significantly differed as a function of empathic disposition, as indexed by high versus low EQ grouping. This test was selected in light of potential heterogeneity in group variances and the slightly unequal sample sizes (n_1_ = 14, n_2_ = 15). The analysis revealed a statistically significant effect of empathic profile on response speed, t(≈26) = –2.52, *p* = 0.018 (two-tailed). Participants in the Low-EQ group exhibited significantly longer RTs (M = 786 ms, SD = 36.7) compared to their High-EQ counterparts (M = 751 ms, SD = 38). The magnitude of the effect, quantified via Cohen’s d, was 0.94, indicating a large effect size, thus suggesting that the observed difference was not only statistically robust but also of substantial practical relevance.

### 3.2. Fast Fourier Frequency Analysis (FFT)

**Figure 2:**
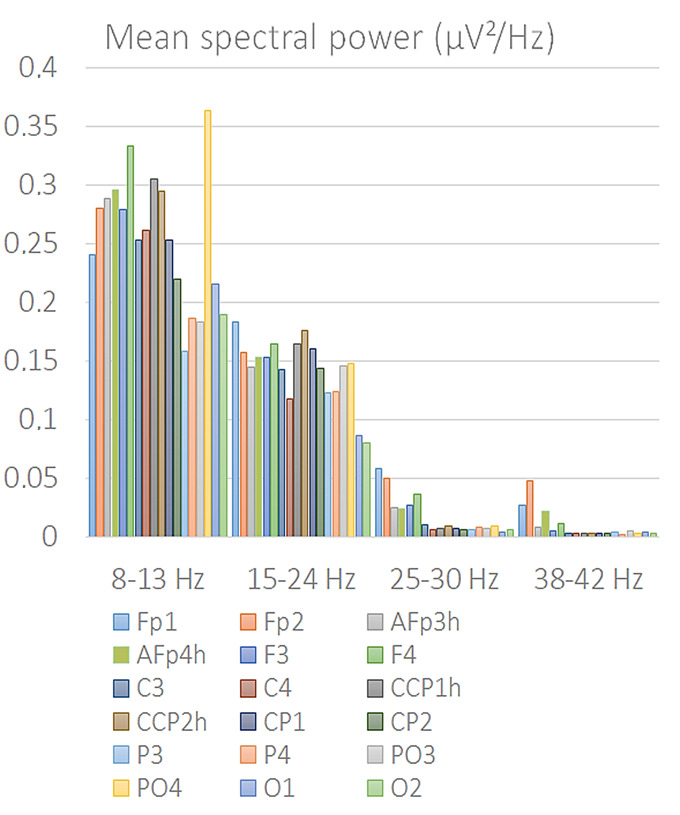
EEG Frequency Analysis performed via Fast Fourier Transform (FFT). Mean spectral power (expressed in µV^2^/Hz) across distinct EEG frequency bands—mu/alpha (8–13 Hz), beta (15–24 Hz), high beta (25–30 Hz), and gamma (38–42 Hz)—for 18 different scalp electrodes located at the anterior, central temporal and posterior brain areas. EEG Frequencies above 25 Hz were excluded from further analyses due to consistently negligible power.

To gain an initial understanding of the spatial distribution of relevant EEG frequency bands, a preliminary FFT analysis was performed. Figure 2 shows EEG Frequency Analysis performed via Fast Fourier Transform (FFT). The line graph illustrates the mean spectral power (expressed in µV^2^/Hz) across distinct EEG frequency bands—mu/alpha (8–13 Hz), beta (15–24 Hz), high beta (25–30 Hz), and gamma (38–42 Hz)—for multiple scalp electrodes. Each point represents the average power computed at a specific electrode site, color-coded accordingly. Notably, the mu/alpha band exhibits the highest power, particularly over parietal-occipital regions, but also medial frontal ones, consistent with typical resting-state EEG topographies. The progressive decline in power from lower to higher frequency bands reflects the expected spectral distribution in cortical activity. Since only alpha/mu and beta ranges showed a significant EEG power, their signals were processed through statistical analyses.

**Figure 3:**
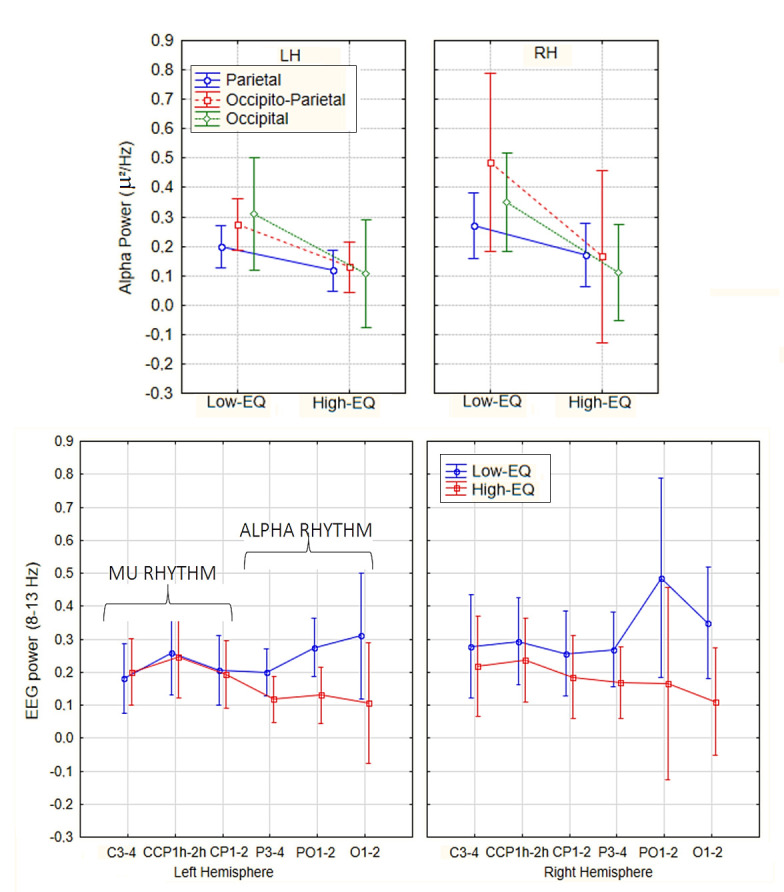
Mean spectral power relative to mu/alpha rhythm (8–13 Hz) according to electrode sites, hemisphere of recording, and empathy group.

An ANOVA conducted on alpha power recorded from posterior scalp sites (P3, P4, PO3, PO4, O1, and O2) revealed a significant main effect of Empathic Trait [F(1, 27) = 4.64, *p* < 0.04], η^2^ₚ = 0.15], with greater alpha power observed in Low-EQ participants (M = 0.315 μV^2^/Hz, SE = 0.06) relative to High-EQ participants (M = 0.13 μV^2^/Hz, SE = 0.05) over occipito-parietal regions (see Figure 3), according to Tukey’s post hoc comparisons (*p* = 0.040). In contrast, no significant main effect of Empathic Trait was found for mu power at central scalp sites (C3, C4, CCP1h, CCP2h, CP1, and CP2), as confirmed via Tukey’s test (*p* = 0.78). However, a significant main effect of Scalp Region emerged [F(2, 54) = 6.10, *p* < 0.005; ε = 0.895; corr. *p* value = 0.006; η^2^ₚ = 0.19], with higher mu power at CCP1 and CCP2 compared to the remaining central sites (*p* < 0.02 and 0.005, Tukey’s test).

**Figure 4:**
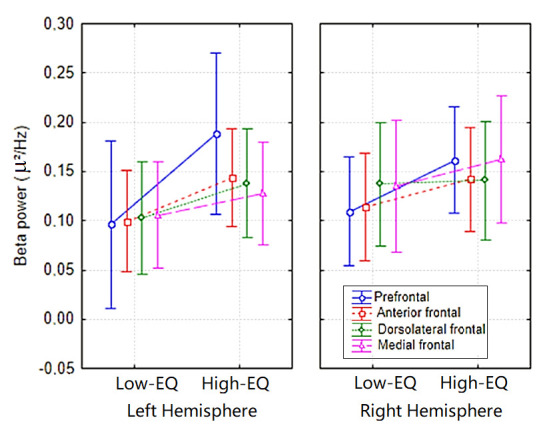
Mean spectral power relative to the beta band (15–24 Hz) according to electrode sites, hemisphere of recording, and empathy group.

**Figure 5:**
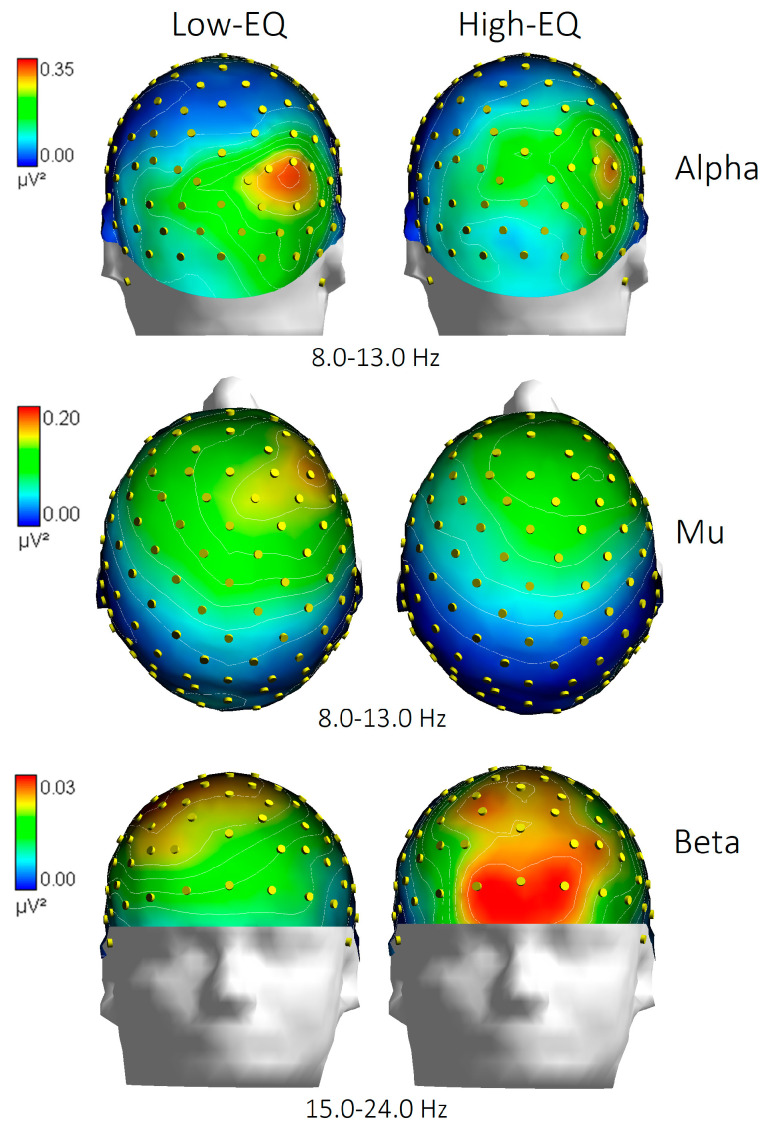
Topographical distribution of EEG spectral power across alpha (8.0–13.0 Hz), mu (8.0–13.0 Hz), and beta (15.0–24.0 Hz) frequency bands for participants with low and high empathy quotient (EQ). Scalp maps illustrate the normalized power (in μV^2^) projected onto a standard head model, with color gradients indicating relative power intensity. Notably, differences between Low-EQ and High-EQ groups emerge prominently in the frontal regions, particularly in the beta band, and in the occipito/parietal regions, particularly in the alpha band.

The analysis of mean beta power at anterior electrodes (Fp1, Fp2, AFp3h, AFp4h, F1, F2, F3, F4) yielded a significant Area × Empathy interaction [F(3, 81) = 3.10, *p* < 0.032; ε = 0.538; corr. *p* value = 0.06; η^2^ₚ = 0.105]. Post hoc comparisons (Duncan’s test = 0.05) indicated greater beta power in High-EQ participants, particularly at prefrontal sites, relative to those with lower trait empathy (see Figure 4 for mean values with SD and Figure 5 for the topographical distribution of all EEG bands).

Given the strong trend toward statistical significance (*p* < 0.03, ε-corrected *p* = 0.06) for the interaction between empathic traits and brain area, we proceeded with more rigorous follow-up analyses to further explore this effect. To investigate whether frontal beta power differed as a function of trait empathy, we extracted beta power from two frontal electrodes (Fp1 and Fp2). For each participant, values were averaged across these electrodes, yielding a single mean frontal beta value. Group differences between low- and High-EQ individuals were evaluated using a two-sided permutation test (10,000 resamples), a robust non-parametric method well suited for EEG data. A trend-level difference in frontal beta activity was observed between groups. Low-EQ participants exhibited lower frontal beta power (M = 0.1027) compared to High-EQ individuals (M = 0.175), although this difference did not reach statistical significance (*p* = 0.0964, two-sided permutation test, 10,000 resamples). To quantify the magnitude of the group difference in frontal beta power despite the lack of statistical significance, we computed Cohen’s d, a standardized effect size metric, based on the mean beta values of the Low- and High-EQ groups. Although the permutation test did not reach conventional significance, the effect size was small to moderate (Cohen’s d = 0.38), indicating a potentially meaningful difference in frontal beta power between individuals with high and low trait empathy.

### 3.3. Wavelet Analysis

**Figure 6:**
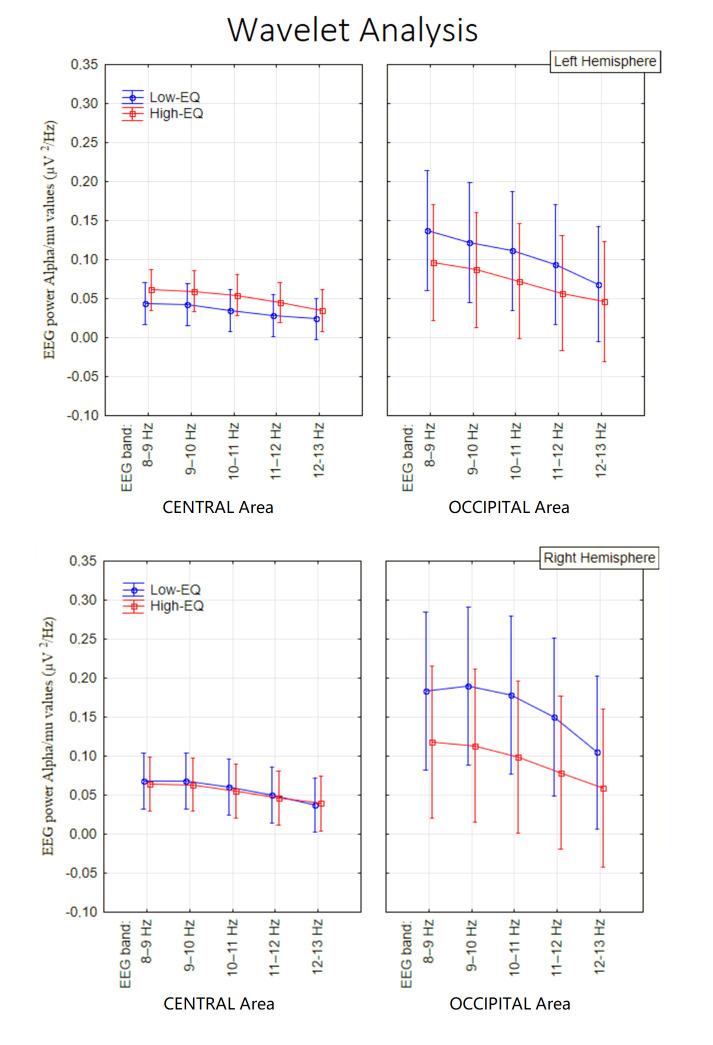
Mean EEG spectral power (µV^2^/Hz) in the alpha/mu range, as a function of electrode site, EEG band, and empathy group.

The ANOVA yielded the significance of Area [F1, 135 = 23.27, *p* < 0.00005; ε = 1; η^2^ₚ = 0.15] with stronger signals over occipital (0.11 µV^2^/Hz, SE = 0.006) than central (0.04 µV^2^/Hz, SE = 0.02) area. Further significant was the factor Area x Empathic trait [F1, 135 = 5.6, *p* < 0.02; ε = 1, η^2^ₚ = 0.04] with greater alpha signals in Low-EQ than High-EQ participants (see Figure 6 for means and SD values) but only over occipital sites (Duncan’s test, *p* = 023; Test Newman–Keuls, *p* = 0.023).

**Figure 7:**
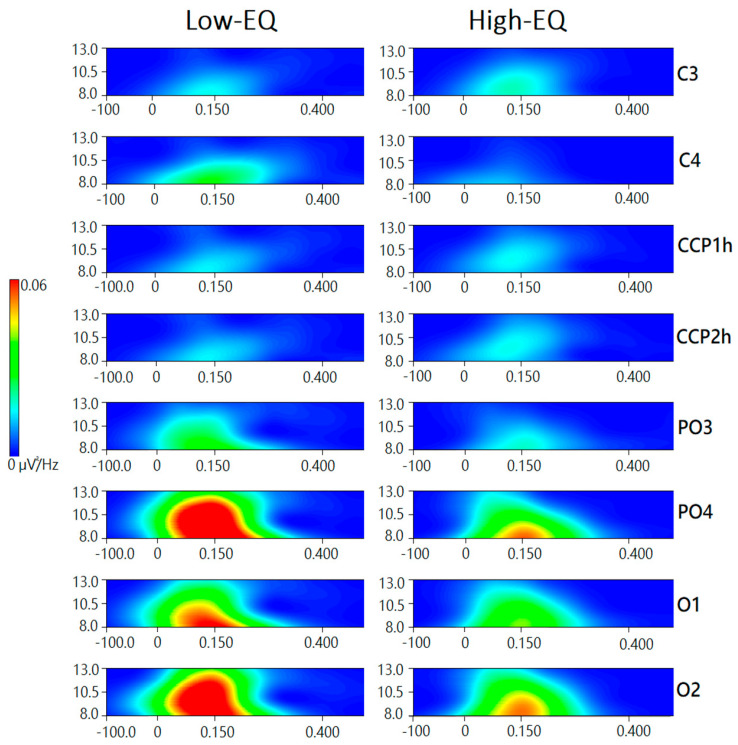
Time–frequency representations of EEG spectral power (µV^2^/Hz) in the 8–13 Hz range for Low-EQ and High-EQ participants, computed using Morlet wavelet transformation. Each subplot displays data from a specific electrode site (central: C3, C4, CCP1h, CCP2h; occipital: PO3, PO4, O1, O2), with time (ms) on the x-axis and frequency (Hz) on the y-axis. Color gradients indicate the magnitude of spectral power, with warmer colors representing higher power. Notable group differences emerge in posterior regions, particularly around 150–250 ms post-stimulus.

No effect of empathy was observed at central sites (mu rhythm). The further significance of Empathic Trait x Hemisphere [F1, 135 = 5.3, *p* < 0.02; ε = 1, η^2^ₚ = 0.041] indicated an enhancement of alpha power over the right hemisphere in Low-EQ participants (Tukey’s test = 0.00001). Also significant was the interaction of Area x Hemisphere [F1, 135 = 11.21, *p* < 0.001; ε = 1, η^2^ₚ = 0.04], with much stronger EEG power over occipital than central area, where a large right hemispheric asymmetry was observed, as shown through Tukey’s post hoc comparisons (hemispheric asymmetry at central area: *p* = 0.09; asymmetry at occipital area *p* = 0.000008) (see Figure 7 for a time–frequency representation, i.e., scalogram).

**Figure 8:**
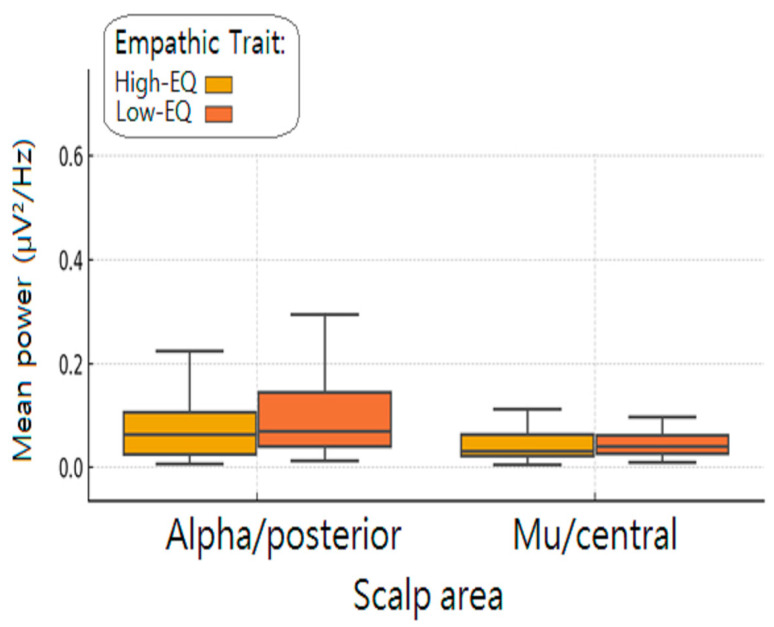
Mean spectral power (μV^2^/Hz) across scalp areas as a function of empathic trait. Boxplots display the average power in the alpha (posterior) and mu (central) frequency bands for participants classified as high or low in empathic traits (High-EQ and Low-EQ). The α-band was measured over posterior scalp regions and the μ-band was measured over central regions. Participants with lower empathic traits (Low-EQ; orange) exhibited increased alpha power in posterior areas compared to high-empathy individuals (High-EQ: yellow). No substantial differences emerged in the mu rhythm across groups. Error bars represent interquartile ranges.

To investigate whether trait empathy modulated oscillatory power in specific cortical regions, we performed targeted post hoc comparisons of Morlet alpha and mu activity between Low- and High-EQ participants. Power values were extracted from two electrode clusters: centro-parietal sites (CCP1h and CCP2h) for the mu rhythm, and occipito-parietal sites (PO3 and PO4) for the alpha rhythm. For each participant, power values were averaged across the selected electrodes and across frequency bins (8–13 Hz), resulting in a single mean value for each region of interest (ROI). Group differences were assessed using two-sided permutation tests with 10,000 resamples. This non-parametric approach does not rely on assumptions of normality and is well suited to EEG data, which often violate parametric assumptions. For each ROI, the distribution of permuted group differences was compared to the observed difference to compute an exact *p*-value. False Discovery Rate (FDR) correction was applied across the two comparisons (mu and alpha) to control for multiple testing (Benjamini–Hochberg procedure). A significant difference in posterior alpha power was observed between groups, with Low-EQ participants exhibiting higher alpha power compared to their High-EQ counterparts (M = 0.152 vs. M = 0.076; *p* = 0.0002, FDR-corrected). This finding is consistent with the interpretation of increased cortical idling or disengagement in individuals with lower trait empathy (Figure 8).

## 4. Discussion

Overall, FFT analyses revealed a trend toward increased anterior beta power and significantly reduced posterior alpha power in High-EQ compared to Low-EQ individuals during a visual task involving body gesture comprehension. Complementary time–frequency analysis using Morlet wavelet transform showed no group differences in mu rhythm suppression, contrary to earlier findings suggesting enhanced mu ERD in high-empathy individuals (e.g., [11,12,13]) as a proxy of mirror neuron activation. Notably, wavelet analysis revealed elevated occipito-parietal alpha power in Low-EQ participants.

### 4.1. Alpha Power (Occipito-Parietal)

Interestingly, Matsuoka et al. [7] demonstrated that alpha-band power in occipito-parietal regions decreases during self-referential and social cognitive tasks, supporting the view that posterior alpha desynchronization reflects increased attentional engagement in empathic contexts. Moreover, increased alpha activity in occipital regions has been linked to cortical inhibition and attentional withdrawal from external stimuli. For instance, Romei et al. [28] showed that greater resting-state alpha power over posterior sites predicted higher visual detection thresholds, indicative of functional inhibition of the visual cortex. These findings collectively support the interpretation that lower alpha power in High-EQ individuals reflects heightened attentional allocation to socially salient visual stimuli. Elevated alpha activity is also frequently associated with diminished vigilance and attentional disengagement. Studies have linked increased alpha power with cognitive fatigue, particularly in the context of sustained attention tasks. For instance, sleep deprivation has been shown to induce alpha bursts and impair vigilance [29], and during prolonged attentional tasks, alpha power tends to increase over time, reflecting declining alertness [30]. Additionally, the observed right-hemispheric asymmetry in posterior alpha power is consistent with prior research indicating that elevated right occipito-parietal alpha is associated with reduced externally directed attention, internally oriented cognition [31], hypoxia-induced attentional suppression [32], and diminished spatial deployment of attention [33].

### 4.2. Frontal Beta Activity

Maffei et al. [34] provided evidence that increased frontal beta activity supports enhanced emotional regulation and attention, which may underlie superior mentalizing abilities in high-empathy individuals. This interpretation is further supported by a growing body of literature linking frontal beta oscillations with top-down attentional control and the modulation of sensory processing [35,36,37]. In this framework, High-EQ participants may allocate more cognitive resources to social information, facilitating the interpretation of body gestures and emotional states. Indeed, individuals with higher empathy quotients tend to exhibit greater interest in others, enhancing their capacity to detect and interpret non-verbal signals [38].

### 4.3. Mu Suppression (or Lack Thereof)

Despite expectations based on previous studies [11,12,13], the present results did not reveal any significant group differences in mu power. The mu rhythm was generally low across all participants, consistent with an event-related desynchronization (ERD) during the processing of body gestures [10,39,40,41,42]. This suggests that core visuomotor mechanisms involved in action understanding may be uniformly engaged across individuals, regardless of empathic disposition. Thus, individual differences in empathy may reflect variability not in mirroring processes per se but in the degree of attentional engagement and motivational relevance attributed to the observed stimuli.

### 4.4. Integration with ERP Findings and Broader Implications

These oscillatory findings are consistent with previous ERP data from the same cohort [21], which indicated that Low-EQ participants exhibited reduced anterior ERP components and poorer task performance in evaluating the emotional, physiological, or mental state of a character. Such differences may reflect diminished attentional engagement with socially meaningful visual cues. Supporting this interpretation, prior studies have shown that individuals with higher empathic traits exhibit greater N170 and LPP amplitudes in response to emotional facial expressions, reflecting increased attention from early perceptual stages [43]. In contrast, lower empathic individuals tend to show diminished neural reactivity, eye scanning, and facial mimicry, consistent with attenuated engagement with emotional content [44].

## 5. Conclusions

In summary, the present findings underscore the role of individual variability in empathic traits in modulating oscillatory brain dynamics during the perception of socially relevant visual cues. High-EQ participants demonstrated a neural profile characterized by enhanced frontal beta activity and attenuated posterior alpha power, suggesting more active top-down attentional engagement in decoding body language. The absence of differential mu suppression further challenges unidimensional accounts of empathy centered on sensorimotor mirroring, pointing instead toward a more integrative model encompassing attentional, regulatory, and motivational components. Importantly, the principal findings were derived from time–frequency analysis using Morlet wavelets, a method known for its sensitivity to transient and non-phase-locked oscillatory activity [19]. While FFT provided complementary spectral information—particularly in the beta band—the most robust group differences, such as increased posterior alpha power in Low-EQ individuals and the lack of mu suppression, emerged via the wavelet-based approach.

Collectively, the findings offer novel evidence that empathy-related neural responses extend beyond mirroring mechanisms and encompass broader attentional and executive control systems. These insights hold important implications for the study of social cognition in both neurotypical and clinical populations and may inform future interventions targeting empathic engagement in educational or therapeutic contexts.

## 6. Limitations and Future Directions

While the present study provides valuable insights into the neural correlates of body language comprehension and trait empathy, several limitations must be acknowledged. First, the relatively modest sample size (N = 30) may limit statistical power, potentially constraining the detection of subtle effects or higher-order interactions. Second, the sample consisted exclusively of university students, which inherently restricts age, educational, and occupational variability. Future research should aim to replicate these findings in larger, more heterogeneous samples to enhance external validity. Third, the assessment of empathy relied solely on self-report measures, which, while widely used and validated, are inherently susceptible to social desirability bias and introspective limitations. To address this, future studies should adopt a multimodal assessment strategy, incorporating behavioral indices (e.g., emotion recognition accuracy and prosocial decision-making tasks) as well as physiological and neural markers, in order to triangulate and enrich the construct validity of empathy-related individual differences. Furthermore, we acknowledge that advanced techniques such as Independent Component Analysis (ICA) and source localization provide critical tools for dissociating overlapping alpha and mu rhythms, particularly in sensorimotor regions. Although these approaches were not implemented in the current study, we fully recognize their potential to enhance the spatial specificity of oscillatory signals and consider them important avenues for future investigations seeking to disentangle the functional roles of distinct frequency components.

## Figures and Tables

**Table 1:** Mean empathy scores for the two participant groups. Normalized scores (obtained by scaling the raw total relative to twice the number of items), minimum and maximum values, and standard deviation (SD) values are also provided. Participants were classified into two groups according to their empathy scores: Low-EQ (range: 0–0.60; raw score ≤ 18) and High-EQ (range: 0.63–1; raw score ≥ 19).

Groups	Norm. Score	N° SS	MIN	MAX	SD	Mean Score
Low-ES	0.51	14	0.27	0.60	0.091	15.28
High-ES	0.73	15	0.63	0.90	0103	21.9

## Data Availability

The stimulus dataset can be inspected at this OA repository: Proverbio, Alice Mado; Gabaro, Veronica (2025), “A Nonverbal Signs Dataset for the Italian Population”, Bicocca Open Archive Research Data, V1, doi:10.17632/vwwmmd99r3.1 [45].

## Abbreviations

The following abbreviations are used in this manuscript:

ANOVA	Analysis of Variance
EEG	Electroencephalogram
EOG	Electro-oculogram
ERD	Event-related desynchronization
ERP	Event-Related Potential
FFT	Fast Fourier Transform
High-EQ	High empathy quotient
Low-EQ	Low empathy quotient
